# Microfluidic Synthesis, Doping Strategy, and Optoelectronic Applications of Nanostructured Halide Perovskite Materials

**DOI:** 10.3390/mi13101647

**Published:** 2022-09-30

**Authors:** Shuangyang Zou, Xiaoan Zhao, Wenze Ouyang, Shenghua Xu

**Affiliations:** 1 Key Laboratory of Microgravity, Institute of Mechanics, Chinese Academy of Sciences, Beijing 100190, China; 2 School of Engineering Science, University of Chinese Academy of Sciences, Beijing 100149, China

**Keywords:** microfluidics, halide perovskite, doping, nanomaterials, optoelectronics

## Abstract

Halide perovskites are increasingly exploited as semiconducting materials in diverse optoelectronic applications, including light emitters, photodetectors, and solar cells. The halide perovskite can be easily processed in solution, making microfluidic synthesis possible. This review introduces perovskite nanostructures based on micron fluidic channels in chemical reactions. We also briefly discuss and summarize several advantages of microfluidics, recent progress of doping strategies, and optoelectronic applications of light-sensitive nanostructured perovskite materials. The perspective of microfluidic synthesis of halide perovskite on optoelectronic applications and possible challenges are presented.

## 1. Introduction

The microfluidic chip that confines fluids in micron channels can scale the chemical reactions from extensive batch synthesis down to the microscale, exploiting the physical and chemical properties of liquids and gases at a microscale, significantly reducing the synthesis and analysis of volume reagents [1,2,3,4,5]. In nanocrystal (NC) synthetic processes, the batch synthesis strategies of NCs are almost always challenging due to rapid perovskite crystallization, the extensive precursor preparation, the difficulties associated with product purification, and the need for particle post-synthesis. It is envisioned that a microreactor platform consisting of flow-focusing microfluidics might be suitable to synthesize high-crystallinity and narrow-size-distribution NCs due to the ultrafast mixing and phase separation during the crystal nucleation and growth. The microfluidic chemical reactions can be precisely detected and explored by in situ spectroscopy [6,7,8,9,10] and more sufficient and continuous during the reaction on the micron scale. Therefore, there are at least two advantages to microfluidic synthesis. On the macroscopic level, a microreactor can be considered a powerful and effective platform for the mass synthesis of semiconductor nanomaterials. On the microscopic level, the microfluidic technique facilitates the simultaneous collection of both absorption and photoluminescence (PL) spectra of various luminescent materials synthesized in the liquid states, particularly that of halide perovskite nanocrystals.

Quantum dot (QD) semiconductors are promising materials for various applications ranging from light-emitting diode (LED) displays to solar cells, biological sensing, and imaging [6,7,8]. Specifically as optoelectronic materials, perovskite nanocrystals have attracted much more attention due to their high PL quantum yields, high absorption/emission efficiency, long carrier lifetime, and tunable emission color over the entire visible region [9,10,11]. Lead halide perovskite structure can be characterized by the general formula ABX_3_ (X = Cl, Br, or I), where A and B represent two different cations. A-site cations can be inorganic or organic ions, such as cesium (Cs), formamidinium (FA), and methylammonium (MA), while B-site cation (Pb^2+^) could potentially be exchanged by dopant ions (Mn^2+^, Fe^2+^, Ce^3+^, Eu^2+^) [12,13,14,15,16,17,18]. Therefore, the hybrid organic−inorganic lead halide perovskite, such as CH_3_NH_3_PbX_3_; and all inorganic lead halide perovskite, such as CsPbX_3_, in the form of nanocrystals, thin films, microcrystals, and bulk single-crystals, show promising properties in LEDs [9,19], lasers [20], solar cells [21,22,23], gas sensors [24], etc. This review will present the development, progress, and perspectives of halide perovskite synthesis and optoelectronic applications.

## 2. Microfluidic Synthesis of Halide Perovskite

**Figure 1:**
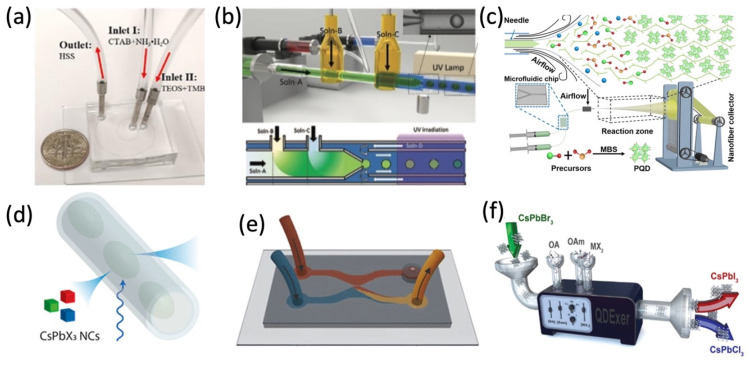
Various microfluidic syntheses of perovskite nanostructures and composite. (**a**) Microfluidic setup with a U.S. dime coin for comparison. Adapted with permission from Ref. [25]. Copyright 2019 Elsevier B.V. (**b**) Synthesis of perovskite composite microparticles. Adapted with permission from Ref. [26]. Copyright 2021 Wiley-VCH GmbH. (**c**) Formation of MAPbBr_3_ PQDs in nanofiber. Adapted with permission from Ref. [27]. Copyright 2022 Wiley-VCH GmbH. (**d**) Schematic of the PL dynamics of microfluidic droplet. Adapted with permission from Ref. [28]. Copyright 2020 American Chemical Society. (**e**) Microfluidic chips for synthesizing CsPbBr_3_. Adapted with permission from Ref. [29]. Copyright 2021 American Chemical Society. (**f**) QD anion exchange reaction in a continuous flow. Adapted with permission from Ref. [30]. Copyright 2019 WILEY-VCH Verlag GmbH & Co. KGaA, Weinheim, Germany.

Generally, microfluidic devices have microchannels ranging from submicron to a few millimeters, as shown in Figure 1, which can move or analyze the tiny amount of liquid (droplet) in a single- or multi-phase flow.

The microfluidic reaction has been recognized to be more controllable and continuous during the nanostructure’s synthesis on the microscale. As shown in Figure 1d, the QDs were synthesized from multiphase (liquid, gas) in microfluidic channels. Compared to conventional flask synthesis under gas protection at high temperatures, the continuous-flow microfluidic approach benefits the alignment of the quantum-confined perovskite nanocrystals and can promote crystal growth orientation to form long nanowires (NWs) at room temperature. In Figure 1a–c,e,f, reagent precursor solution is injected into the microchannel. After combination in the channel, different types of nanomaterials can be achieved (Table 1) [25,26,27,29,31,32,33].

**Figure 2:**
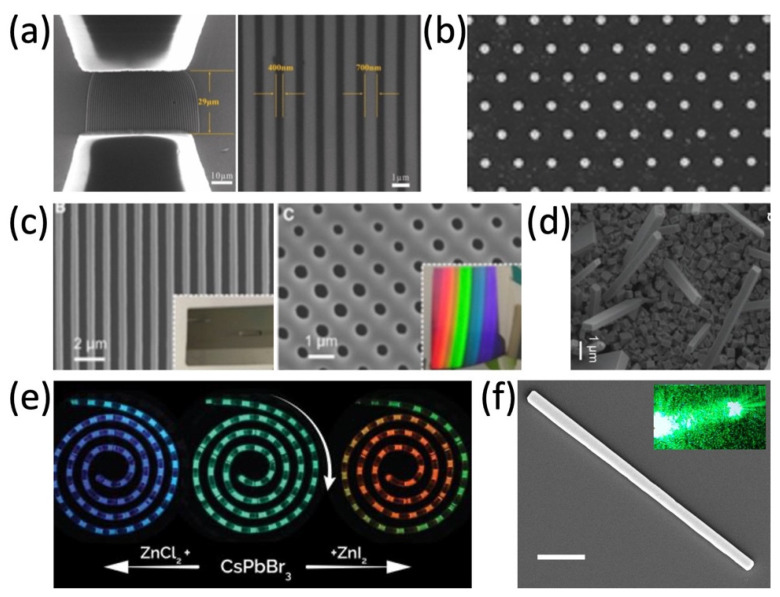
(**a**) SEM images of the silicon nanowire template for microfluidic synthesis. Adapted with permission from Ref. [45]. Copyright 2020 The Royal Society of Chemistry. (**b**) SEM image of the CH_3_NH_3_PbX_3_ platelet array. Adapted with permission from Ref. [43]. Copyright 2016 American Chemical Society. (**c**) Periodic parallel lines and surface of grating-patterned Si substrate, respectively. Adapted under a creative commons license from Ref. [46] (www.creativecommons.org/licenses/by-nc-nd/4.0/ (accessed on 14 September 2022)). Copyright 2020 The Authors. (**d**) SEM image of the CsPbI_3_ NWs. Adapted with permission from Ref. [44]. Copyright 2016 American Chemical Society. (**e**) Continuous anion exchange reactions of CsPbBr_3_ QDs. Adapted with permission from Ref. [30]. Copyright 2019 WILEY-VCH Verlag GmbH & Co. KGaA, Weinheim. (**f**) SEM image of the Fe-doped CsPb(Cl/Br)_3_ NW. Adapted with permission from Ref. [17]. Copyright 2018 American Chemical Society.

The microfluidic channel with controllable morphology and configuration could be efficiently designed and achieved, therefore, nanomaterials could be more precisely synthesized in the microfluidic channel. For example, Kim et al. reported the in situ reaction of metal halide perovskite nanoparticles by the ligand-assisted reprecipitation process (LARP) and encapsulation by ultraviolet light (UV) cross-linking polymerization, in which the stable, water-resistant light-emitting perovskite–polymer composite microparticles can be synthesized in a continuous one-step microfluidic reactor [26]. Tuning the reactant concentration and the flow rate in the microreactor, ranging from several nanometers to over one hundred nanometers, hollow spherical silica-based functional materials and the Cs_4_PbBr_6_ perovskite microcrystals (MCs) were synthesized by mixing two reactant flows, respectively [25,37]. With the microfluidic template in Figure 2a,c, well-aligned and uniform heterojunctions of MAPbI_3_ and organic semiconductors (OSC) in the silicon nanowire patterns can be grown. In Figure 2b,d,f, different morphologies (1D, 2D) of halide perovskite have already been successfully synthesized via solution methods [17,43,44], which are difficult to batch produce and industrially apply in comparison to microfluidic synthesis. In Figure 2e, the halide exchange reactions are realized in a modular microfluidic platform called Quantum Dot Exchanger, which offers a unique time- and material-efficient approach for studies of solution phase-processed colloidal nanocrystals [30,45,46]. Perovskite precursor solutions could be simultaneously pumped into the microfluidic device. By changing the ratio of different perovskite precursor solutions, a series of perovskite QDs can be precipitated and encapsulated in ethyleneglycol dimethacrylate (EGDMA) resin [32]. The microfluidic synthesis makes chemical composition tuning and doping in perovskite more available.

## 3. Doping Strategies

### 3.1. Ion Doping

Much research on structural design and optical properties of semiconductors has recently been studied extensively concerning defects, isovalent and aliovalent doping [12,47,48,49]. Chemical doping of halide perovskite is a promising strategy to prepare the highest efficiency and most stable perovskite-based devices [50,51]. The doping ions can be alkali metals (K^+^), alkaline earth metals (Sr^2+^, Mg^2+^), transition metal ions (Mn^2+^, Fe^3+^), lanthanide ions (Ce^3+^, Nd^3+^, Eu^2+^), etc. [15,16,17,18,52,53]. However, it is still a challenging step for controllable doping in halide perovskite family of semiconductors, due to compensation from and facile migration of intrinsic defects [54].

**Figure 3:**
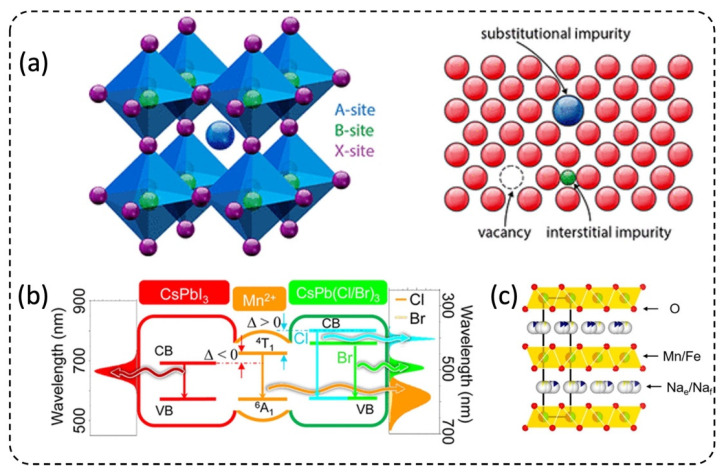
Structure properties of doping. (**a**) Basic ABX_3_ crystal structure and possible dopant locations of perovskite semiconductor. Adapted with permission from Ref. [51]. Copyright 2021 American Chemical Society. (**b**) The energy level diagram of Mn-doped CsPbX_3_ NCs. Adapted with permission from Ref. [16]. Copyright 2016 American Chemical Society. (**c**) Schematic of layered crystal structure. Adapted under a creative commons license from Ref. [58] (www.creativecommons.org/licenses/by-nc-nd/4.0/ (accessed on 14 September 2022)) Copyright 2021 by the authors.

A dopant is often used to retain the material’s morphology while partially changing its composition (Figure 3a), exhibiting a distinct difference that is not otherwise attainable in a crystalline host material, such as carrier concentration, luminescence centers, bandgap tuning, and excitons [16,55,56]. B-site doping (metal substitution) in perovskite is more likely to enable tuning of carrier concentration and Fermi level [51]. For example, a small amount of bismuth dopant in tin iodide cubic perovskite affects the electronic structure and electronic properties of this material, which causes the continued narrowing of the band gap from 1.3 to 0.8 eV without changing the energy and density of states (DOS) at the top of the valence band, and without increasing the number of carriers [57]. Phung et al. unveiled the alkaline earth metals (Sr^2+^, Mg^2+^) doping mechanism: low doping levels enable the incorporation of the dopant within the perovskite lattice, whereas high doping concentrations induce surface segregation [52]. Figure 3b demonstrates the Mn emission band in transition metal cation Mn^2+^-doped perovskite NCs [16]. Moreover, the dopant in the crystal structure also leads to slightly improved electrochemical performances, such as discharge capacity and rate capability (Figure 3c) [58,59].

The merits of microfluidics in doping perovskite are the efficient mixing of the precursor ions, the rapid nucleation of crystal seeds in the antisolvent, and the controllable crystal growth of the doped perovskite along the flow direction, which may further improve the quality and quantity of dopant in halide perovskite. Several dopants in perovskite have been successfully realized in the microfluidic reactor, such as lanthanide ions in CsPbBr_3_ perovskite. Lin et al. investigated the Ce^3+^ concentration effect on PL efficiency, quantum yield, and perovskite stability at ambient conditions [41]. Integrating in situ spectral characterizations with the modular microfluidic platform is an advantage to rapidly investigating the precursor concentration and ligand migration kinetics and accurately revealing the doping mechanism of perovskite QDs [60].

### 3.2. Ion Exchange

The ion exchange for halide perovskite refers to the progress that the ions in the reaction exchange with the counterpart of the parent crystalline compound to form a crystal lattice with entirely or partly exchanged ionic components [61]. Microfluidic synthesis could increase the efficiency of ion exchange at low temperatures. For example, Abdel- Latif et al. reported the effects of ligand composition and halide salt source on room-temperature, single-solvent anion exchange reaction kinetics and bandgap properties with CsPbBr_3_ perovskite QD solution using optical spectroscopy [30]. With the combination of online photoluminescence and absorption measurements and the fast mixing of reagents in a microfluidic platform, Lignos et al. reported the rigorous and rapid mapping of the reaction parameters of CsPbX_3_ nanocrystals, including the effects of molar ratios of Cs, Pb, and halide precursors, reaction temperatures, and reaction times [38]. Via controlled anion exchange reactions using a range of different halide precursors, Akkerman et al. demonstrated the tunable chemical composition and the optical properties of colloidal CsPbBr_3_ NCs in the region of the visible spectrum by displacement of Cl^−^ or I^−^ ions and reinsertion of Br^−^ ions [62]. The microfluidic platform can potentially and comprehensively understand the halide exchange reactions by tuning precursor mixing rates in the microfluidic channel.

## 4. Optoelectronic Applications

### 4.1. LEDs and Laser

Halide perovskite used as a photoactive layer has been widely explored in optoelectronics, e.g., LEDs, lasers, photodetectors, solar cells, etc., due to high quantum yields and tunable light emission [9,10,63]. Several light-sensitive perovskite devices were fabricated based on microfluidic synthesis.

**Figure 4:**
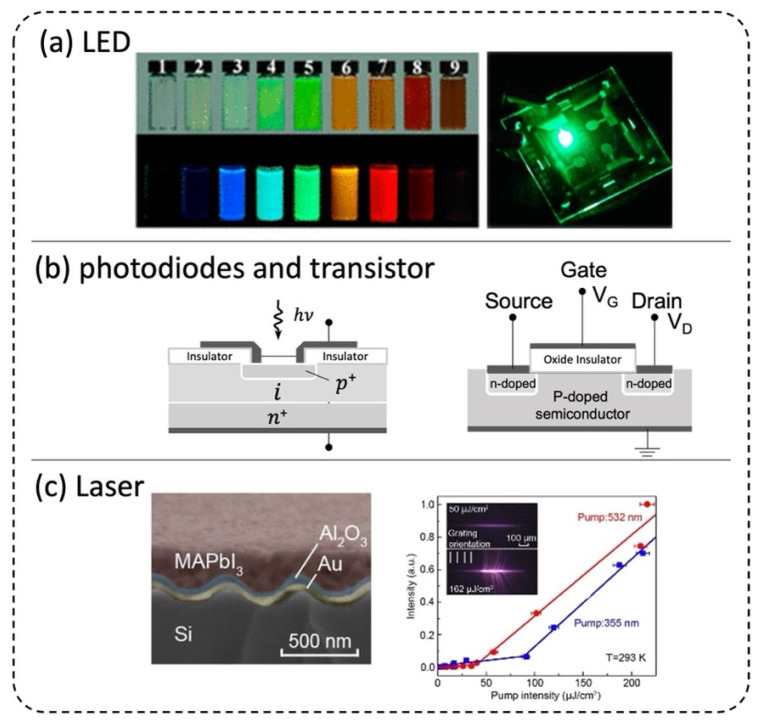
Optoelectronic applications of perovskite. (**a**) Color-tunable CH3NH3PbX3 (X = Cl, Br, or I) QDs and perovskite LEDs. Adapted with permission from Refs. [10,64]. Copyright 2020 American Chemical Society and 2017 American Chemical Society, respectively. (**b**) Schematic diagram of 𝑝-𝑖-𝑛 photodiodes and MOSFET. (**c**) Cross-sectional profiles and threshold of MAPbI3 DFB laser. Adapted with permission from Ref. [67]. Copyright 2016 American Chemical Society.

Perovskite LEDs have achieved impressive progress in the past few years (Figure 4a), showing that quantum efficiency has surpassed 20 per cent for managed compositional distribution and balanced charge injection [63,64]. Using a microfluidic system, Cs_4_PbBr_6_ perovskite MCs were fabricated with K_2_SiF_6_:Mn^4+^ phosphor onto InGaN blue chips as white LEDs, which achieved a high National Television Standards Committee value of 119% for backlight display [37]. The Ce^3+^-doped CsPbBr_3_ perovskite NCs were used to manufacture the green LEDs with a high color purity of 93.3% and the white LEDs [41]. Due to the availability of nanoreactors for chemical synthesis with scale-up capacities, large-scale production of ligand-free (MAPbX_3_, X = Cl, Br, and I) perovskite QDs has been realized with a microfluidic blow spinning technique. The composite nanofiber film production (120 cm × 30 cm per hour) exhibited a high color gamut of 126.2% [27], potentially useful for wide-color-gamut displays and LEDs. 

Perovskite lasers are promising light sources with great potential for integration into photonic circuits. Over the past few years, many types of perovskite lasers have been demonstrated, e.g., Fabry-Perot, DBR, DFB, etc. [20,65]. By designing chip configuration and reagents flow rates in a microfluidic chip, one can fabricate the controllable morphology of CsPbBr_3_ NWs lasers in the form of suspension obtained by rapid precipitation, which can be deposited on an arbitrary surface [29]. The single crystalline NWs with smooth end facets and subwavelength dimensions are ideal Fabry–Pérot cavities for NW lasers. Fu et al. demonstrated optically pumped tunable F–P lasing across the entire visible spectrum (420–710 nm) from NW at room temperature [44]. A vertical-cavity surface-emitting perovskite laser was achieved with a morphologically highly uniform CH_3_NH_3_PbI_3_ perovskite thin film placed between two high-reflectivity GaN-based distributed Bragg reflectors (DBRs). This single-mode perovskite laser reaches a low threshold (~7.6 µJ cm^−2^) at room temperature and emits spatially coherent Gaussian laser beams. [66] As shown in Figure 4c, Jia et al. demonstrated metal-clad MAPbI_3_ distributed feedback (DFB) lasers that operate at a pump intensity threshold of 5 kW/cm^2^ for durations up to ~25 ns under InGaN diode laser excitation at low temperature, which indicates the potential electrically pumped lasing [67,68]. Shang et al. demonstrated CW-pumped lasing from one-dimensional CsPbBr_3_ nanoribbons (NBs) with a threshold of ∼130 W cm^−2^ [69]. The refractive index and the exciton–polariton (EP) effect on continuous-wave (CW) optically driven lasing have been discussed. Optically pumped continuous-wave (CW) lasing [70,71] of perovskite is being researched as gain media will be a prerequisite for electrically pumped perovskite laser in the future.

### 4.2. Photodetectors

The transistor forms the basis of modern electronic integrated circuits, while 𝑝-𝑖-𝑛 junctions of photodiodes could have the function of separating electrical transportation and optical sensitization (Figure 4b), e.g., a hybrid phototransistor and photojunction field-effect transistors (photo-JFETs). A transport channel is formed and modulated by an external gate voltage (Vg) and light illumination. Under illumination, charges are generated in the photoactive material. Depending on the gate voltage, photo-induced charge carriers can be injected into the transporting medium and recirculate several times before recombination, thus producing gain under illumination [72]. The structure of phototransistors has been reported in hybrid perovskite and all-inorganic perovskite. For example, Xin et al. fabricated a cost-effective photodetector consisting of the well-aligned parallel CsPbBr_3_ perovskite MW arrays confined in the Si microchannels [46]. The microwire arrays have good responsivity and may be feasible for large-scale perovskite-based applications. Moreover, flexible substrates have several advantages over rigid glass substrates, which are suitable for portable and wearable device requirements. Based on a microfluidic channel, a lateral structure MAPbI_3_ phototransistor with mobility calculated to be ~1.7 cm^2^ V^−1^ s^−1^ on ITO-coated flexible PET substrate is reported by Khorramshahi et al. [35].

### 4.3. Solar Cells and Sensor

Microfluidic processing has been also utilized in perovskite solar cells (PSCs). Perovskite films received a boost in photovoltaic efficiency through the controlled formation of charge-generating films and improved current transfer to the electrodes. Zhou et al. lowered the defect density of the film by controlling humidity while the perovskite film formed from lead chloride and methylammonium iodide. Low-temperature processing steps allowed the use of materials that draw current out of the perovskite layer more efficiently and have a maximum cell efficiency of over 19% [23]. Michalska et al. demonstrated that the microfluidic-mixing enhanced hole-transporting layers exhibit dramatic reductions in surface energy and an increase in hole mobilities in PSCs, the highest PSCs efficiency up to 15.9% [73]. Besides, based on the PL spectral shifts of perovskite nanocrystals, using perovskite CsPbX_3_ (X = Cl, Br, or I) nanocrystals as a nanoprobe, a paper-based microfluidic sensor through anion exchanging was developed to achieve convenient detection of haloalkanes (CH_2_Cl_2_, CH_2_Br_2_) [24].

## 5. Conclusions and Perspectives

Halide perovskites possess outstanding optical characteristics that can be potentially employed in optoelectronics fabrication, from lasers to solar cells. The perovskite QDs are mainly synthesized by the traditional hot injection method. In comparison, microfluidic synthesis has several advantages: very small quantities of samples and reagents, high resolution and sensitivity in detections, and continuous reaction for scalable synthesis. A-site and B-site doped perovskite can be synthesized in the confined micro-channel based on continuous flow, which is expected to simplify the synthesis process significantly and reduce QDs’ costs. However, several issues should receive much attention in future works. Firstly, although the microfluidic synthesis has successfully demonstrated perovskite QDs patterned structures, the anisotropic growth mechanism should be further investigated. Secondly, there is still plenty of room for the epitaxy growth of perovskite nanostructure at low temperatures. The reaction environment provided by microfluidic facilitates the chemical defect engineering of quantum dot heterostructures. More work is needed to combine physical models to describe the dopant diffusion during the perovskite-growing process. Finally, low-temperature reaction conditions can expand the selectivity of precursors and ligands for green synthesis. We believe microfluidic synthesis has increased the diversity of nanostructured perovskite preparation and doping strategies and will potentially facilitate optoelectronic applications of nanostructured perovskite materials.

## Figures and Tables

**Table 1:** Microfluidic synthesis of nanostructured halide perovskite.

Materials	Synthesis Temp. (°C)	Size	PL Peak Location (nm)	Year [Ref.]
CsPbBr_3_ QDs	RT	<10 nm	~500	2017, Epps et al. [33]
CsPbBr_3_ QDs	RT	10–20 nm	~520	2019, Wei et al. [34]
CsPbBr_3_ NWs	50	3–9 μm	535	2021, Koryakina et al. [29]
CsPbBr_3_ NWs	50	~4 nm (width)	~475	2019, Zhang et al. [31]
MAPbI_3_	85	60 μm (width)	-	2020, Khorramshahi et al. [35]
QD encapsulation	37	500–700 nm	430–625	2021, Bian et al. [32]
MAPbBr_3_ composite	-	500 μm	~530	2021, Kim et al. [26]
FAPb(I/Br)_3_ QDs	120	~10 nm	530–690	2017, Maceiczyk et al. [36]
Cs_4_PbBr_6_ MCs	60–150	>1 μm	520	2018, Bao et al. [37]
CsPbX_3_ QDs	130–220	8–12.5 nm	470–690	2016, Lignos et al. [38,39,40]
CsPbX_3_ QDs	RT	<20 nm	422–660	2019, Abdel-Latif et al. [30]
CsPbX_3_ NCs	RT	~15 nm	~520	2020, Lin et al. [41]
CsPbX_3_ NCs	100–180	<20 nm	406–677	2022, Geng et al. [42]

In the materials column of the table, QDs: quantum dots, NCs: nanocrystals, NWs: nanowires, MCs: microcrystals, and X = Br, I, Cl, respectively.
